# Functional Studies of T Regulatory Lymphocytes in Human Schistosomiasis in Western Kenya

**DOI:** 10.4269/ajtmh.17-0966

**Published:** 2018-04-23

**Authors:** Bartholomew N. Ondigo, Eric M. Ndombi, Sarah C. Nicholson, John K. Oguso, Jennifer M. Carter, Nupur Kittur, W. Evan Secor, Diana M. S. Karanja, Daniel G. Colley

**Affiliations:** 1 Centre for Global Health Research, Kenya Medical Research Institute, Kisumu, Kenya;; 2 Department of Biochemistry and Molecular Biology, Egerton University, Nakuru, Kenya;; 3 Department of Pathology, Kenyatta University, Nairobi, Kenya;; 4 Center for Tropical and Emerging Global Diseases, University of Georgia, Athens, Georgia;; 5 Division of Parasitic Diseases and Malaria, Centers for Disease Control and Prevention, Atlanta, Georgia;; 6 Department of Microbiology, University of Georgia, Athens, Georgia

## Abstract

Immunoregulation is considered a common feature of *Schistosoma mansoni* infections, and elevated levels of T regulatory (Treg) lymphocytes have been reported during chronic human schistosomiasis. We now report that the removal of Treg (CD4+/CD25^hi^/CD127^low^ lymphocytes) from peripheral blood mononuclear cells (PBMCs) of *S. mansoni*–infected individuals leads to increased levels of phytohemagglutinin (PHA)-stimulated interferon gamma (IFNγ) production and decreased interleukin-10 (IL-10) responses. Exposure to schistosome antigens did not result in measurable IFNγ by either PBMC or Treg-depleted populations. Interleukin-10 responses to soluble egg antigens (SEA) by PBMC were unchanged by Treg depletion, but the depletion of Treg greatly decreased IL-10 production to soluble worm antigenic preparation (SWAP). Proliferative responses to PHA increased upon Treg removal, but responses to SEA or SWAP did not, unless only initially low responders were evaluated. Addition of anti-IL-10 increased PBMC proliferative responses to either SEA or SWAP, but did not alter responses by Treg-depleted cells. Blockade by anti-transforming growth factor-beta (TGF-β) increased SEA but not SWAP proliferative responses by PBMC, whereas anti-TGF-β increased both SEA- and SWAP-stimulated responses by Treg-depleted cultures. Addition of both anti-IL-10 and anti-TGF-β to PBMC or Treg-depleted populations increased proliferation of both populations to either SEA or SWAP. These studies demonstrate that Treg appear to produce much of the antigen-stimulated IL-10, but other cell types or subsets of Treg may produce much of the TGF-β. The elevated levels of Treg seen in chronic schistosomiasis appear functional and involve IL-10 and TGF-β in antigen-specific immunoregulation perhaps leading to regulation of immunopathology and/or possibly decreased immunoprotective responses.

## Introduction

Schistosomiasis is a common neglected tropical disease that affects more than 230 million people worldwide.^1^ Multiple studies have described major roles for immune responses with regard to both morbidity and resistance to reinfection in human schistosomiasis.^2–7^ T Regulatory (Treg) lymphocytes are unique subpopulations of T cells involved in immune homeostasis and tolerance^8–11^ and their elevation has been reported in human schistosomiasis.^2,4,12,13^ Regulation of effector T cells during chronic antigenic exposure, such as in schistosomiasis, may protect the host from excessive pathology, but may also impair effective immune-mediated resistance to reinfection. However, Treg quantification and functionality during disease states remains controversial, in part because of the state of flux of reliable markers and the challenge of antigen-specific and nonspecific functional assays.^14,15^ In this study we have further characterized Treg from individuals with schistosomiasis and evaluated the functional capabilities of their Treg in regard to schistosome antigen-specific and mitogen-stimulated proliferative and cytokine responses.

## Methodology

### Study area and study population.

This study was done in Kisumu County in western Kenya. Kisumu is located on the shores of Lake Victoria, where transmission of *S. mansoni* is well documented.^13,16–18^ The study participants (median age 31 years, range 18–63 years) were men employed as sand harvesters or car washers, occupations that expose them daily to schistosome transmission in Lake Victoria.^16,18^

### Ethical considerations.

The objective of the study was explained to all study participants and written consent was obtained from each subject. The research protocol was approved by the Scientific Steering Committee of the Kenya Medical Research Institute (SSC-KEMRI), KEMRI/Scientific Ethical Review Unit (Protocol No. 1913) and the Institutional Review Boards at the University of Georgia (Protocol No. 00004080) and the Centers for Disease Control and Prevention. Centers for Disease Control and Prevention investigators were determined to be nonengaged from a human subject perspective.

### Fecal examinations for helminth parasites.

Infection by *S. mansoni*, *Ascaris lumbricoides*, *Trichuris trichiura*, and hookworm was determined by Kato–Katz fecal examination based on three consecutive stools, two slides each.^19^ The intensity of the infection was obtained for *S. mansoni* as eggs per gram of feces (EPG) and the presence or absence of eggs of the three soil-transmitted helminths (STH) was recorded. The intensity of *S. mansoni* infection was classified according to the World Health Organization (WHO 2013) guidelines^20^ as light (1–99 EPG), moderate (100–399 EPG), and heavy infections (≥ 400 EPG). Individuals positive for *S. mansoni* were treated with 40 mg/kg Praziquantel (PZQ) and anyone positive for STH was treated with 400 mg albendazole.

### Peripheral blood mononuclear cell (PBMC) immunophenotyping, isolation, and Treg depletion.

Heparinized blood (8–20 mL) was collected by sterile venipuncture after diagnosis by Kato–Katz assay and before treatment with PZQ, and immunophenotyping was performed using the following antibodies: anti-CD3, anti-CD4, anti-CD25, anti-CD127, and anti-FOXP3 directly labeled with FITC, PE, PerCP, APC or AF 488. Anti-CD3 (AF 488, PerCP or PE conjugated) clone, UCHT1; anti-CD4 (AF 488 or PerCP conjugated) clone, OKT-4; anti-CD4 (AF488 or APC conjugated) clone, RPA-T4; anti-CD25 (APC or PE conjugated) clone, BC96; anti-CD25 PerCP conjugated, clone, M-A251; anti-FOXP3 PerCP AF488 conjugated, clone, 206D. All the aforementioned reagents were purchased from BioLegend (San Diego, CA). Anti-CD127-APC (Clone: eBioRDR5) was purchased from eBioscience (San Diego, CA). Intracellular FOXP3 staining was performed using the Human FoxP3 Buffer Set (Becton Dickinson, Franklin Lakes, NJ) according to the manufacturer’s instructions. Data were collected using a four-color FACSCalibur^™^ flow cytometer (BD Biosciences, San Jose, CA) and analyzed using FlowJo software version 10.1 (TreeStar, Ashland, OR). Gating was done using the fluorescence minus one procedure as reported previously in detail.^13^

Peripheral blood mononuclear cells were obtained by density gradient centrifugation of heparinized venous blood over Fico/Lite (Atlanta Biologics, Atlanta, GA) within 2 hours of collection. Peripheral blood mononuclear cell were split into two aliquots consisting of ≥ 5.0 × 10^6^ cells each and either not processed further or processed for depletion of CD25^hi^ cells using anti-CD25 magnetic beads (Miltenyi Biotec, Bergisch Gladbach, Germany). Briefly, CD25 + cells were magnetically labeled with 40 μL of CD25 MicroBeads II. The cell suspension was then loaded onto a MACS^®^ Column (Miltenyi Biotec), which was placed in the magnetic field of a MACS Separator. The flow-through containing unlabeled cells was collected (CD25 Treg-depleted population). Following the Treg depletion process, aliquots of both unprocessed PBMC and Treg-depleted populations were analyzed for expression of CD3, CD4, CD25, and CD127 as described above.

### Parasite-derived antigens and phytohemagglutinin (PHA).

Soluble egg antigens (SEA) and soluble worm antigenic preparation (SWAP) from *S. mansoni* were prepared as previously described^21,22^ and titrated at 1, 2.5, 5, and 10 μg/mL of culture medium to determine the optimal concentrations for cell culture stimulation. Antigen stimulation for cultures was optimized at a final concentration of 5 μg/mL for each of the schistosome antigen preparations. Phytohemagglutinin was used at a final concentration of 2.5 μg/mL of culture medium.

### Evaluation of mitogen or antigen-specific cytokine production.

Peripheral blood mononuclear cell and Treg-depleted populations were cultured in 96-well round-bottom plates at a concentration of 2 × 10^5^ cells per well in 200 μL of complete medium RPMI 1640 (Gibco Life Technologies, Grand Island, NY); 10 mM Hepes buffer (Sigma-Aldrich, St. Louis, MO); 10 mM L-glutamine (Sigma-Aldrich); 1% penicillin/streptomycin (Sigma-Aldrich); and 5% human serum (Sigma-Aldrich). The cells were exposed to SEA or SWAP at final concentrations of 5 μg/mL, PHA at a final concentration of 2.5 μg/mL (Sigma-Aldrich) or cultured in complete medium alone as an unstimulated control. Cultures were performed in triplicate or quadruplicate and incubated for either 72 hours (PHA) or 120 hours (SEA or SWAP) at 37°C with humidity and 5% CO_2_; the supernatant fluids were harvested and stored at −20°C for cytokine testing.

### Cytokine enzyme-linked immunosorbent assays (ELISA).

Cytokines (IL-10 and IFN**γ**) were measured in culture supernatant fluids by enzyme-linked immunosorbent Duo-kit assays according to the manufacturer’s protocol (R&D Systems, Minneapolis, MN). Briefly, 96-well flat-bottom plates (Immulon 2 HB; Dynex Technologies Inc., Chantilly, VA) were coated with 100 µL of a capture antibody diluted in phosphate-buffered saline (PBS; wash buffer) and incubated overnight at 4°C. The plates were washed three times with PBS plus 0.05% Tween 20 (Sigma-Aldrich), and nonspecific binding was blocked with PBS plus 1% bovine serum albumen at 300 µL per well for 1 hour. Plates were then washed three times and cytokine standard curves were prepared with cytokine standards serially diluted from 0 to 1,000 pg/mL or 0 to 2,000 pg/mL for IFNγ and IL-10, respectively. The specimens, diluted 1:1 in RPMI to ensure the values fell on the linear portion of the standard curves were added at 100 µL per well followed by 2 hours incubation at room temperature (RT). Plates were then washed thrice with wash buffer and the appropriate biotinylated anti-cytokine antibody was added for another 2- hour incubation at RT. This was followed by another three washes before addition of streptavidin-horseradish peroxidase conjugate solution and incubation for 20 minutes at RT. After a final three washes, the plates were developed with Tetramethylbenzidine peroxidise substrate for approximately 15 minutes, stop solution (2 N sulfuric acid) was added (50 μL/well) and the optical density (O.D.) of each well determined immediately using a Spectramax Emax plate reader (Molecular Devices, Sunnyvale, CA) at 450 nm. Cytokine detection limits were 2 pg/mL (for IL-10) and 8 pg/mL (for IFN-γ). None of the samples tested went through more than one freeze–thaw cycle before being assayed for cytokine levels.

### BrdU proliferation assay.

To measure lymphocyte proliferation, cells (2 × 10^5^) were either cultured in the presence of PHA and media control or *S. mansoni* antigens (SEA and SWAP) and media control, for 3 and 5 days respectively, as detailed previously for cytokine production except cultures were 200 μL in volume in flat-bottom 96 well plates (CoStar, Corning, NY). Proliferation was measured by quantifying DNA levels at the time of harvest using BrdU kits per the manufacturer instructions (Roche Diagnostics, Mannheim, Germany). Therefore, for the last 16 hours of incubation, 20 μL/well of BrdU labeling solution was added to each well. On harvesting, the labeling medium was removed by centrifuging the culture plate at 300 g for 10 minutes followed by flicking the media off the plate. Cells were fixed and DNA denatured by the addition of 200 μL/well FixDenat that was provided with the kit. This was followed by the incubation of the plate for 30 minutes at RT. The FixDenat solution was then removed by flicking and tapping. Anti-BrdU-POD (100 μL/well 1:100 dilution) was added to each well and incubated for 90 minutes at RT. Anti-BrdU was removed and the wells rinsed three times with 300 μL/well of wash buffer. Subsequently, 100 μL/well of substrate was added and the plate incubated for 5 minutes at RT. The reaction was stopped by the addition of 25 μL/well of 1 M H_2_SO_4_ and absorbance was read at 450 nm on a Spectramax Emax plate reader (Molecular Devices).

### Potential impact of anti-IL-10 and/or anti-TGF-β on antigen-stimulated proliferation.

To evaluate the effect of blockade of IL-10, TGF-β or both IL-10 and TGF-β on proliferation assay cultures exposed to either SEA or SWAP, cultures of PBMC or Treg-depleted populations were incubated in parallel with 20 μL (100 μg/mL) of anti-IL-10 monoclonal antibody (mAb), clone: JES3-9D7; 20 μL (100 μg/mL), antihuman TGF-β mAb clone: 19D8; or irrelevant isotype-control mAbs (purified rat IgG1 clone: RTK2071; purified mouse IgG1, clone: MOPC-21) for each of the anti-cytokine mAbs. All mAbs were from BioLegend and were added to make a final concentration of 10 μg/mL in culture.

### Statistical analysis.

Data were entered into Microsoft Access 2010 databases. Individual datasets were generated using IBM SPSS Statistics for Windows, Version 24.0 (IBM Corp., Armonk, NY). GraphPad Prism version 6 for windows (GraphPad Software, San Diego, CA) was used for statistical analyses and for preparing graphs. Correlations between lymphocyte populations were examined using Spearman’s correlation test. Differences in cytokine production and proliferative responses between total and depleted lymphocytes were evaluated using the Wilcoxon matched-pairs signed rank test. Tests were considered statistically significant at *P* < 0.05.

## Results

### Epidemiological and demographic characteristics of the study participants.

The study participants were at high risk of acquiring schistosomiasis due to occupational exposure as either sand harvesters or car washers who work in shallow water along the shores of Lake Victoria. Essentially, all sand harvesters (*N* = 33) are lifelong residents of a village endemic for *S. mansoni* and have been persistently exposed since they were infants. More than 85% of the car washers (*N* = 45) were initially exposed to possible transmission of *S. mansoni* as adults, when they began their employment as car washers.^16^ At the time of blood sample collection for these studies, the arithmetic mean intensity of *S. mansoni* infection for sand harvesters was 374 EPG (range 8–2,309) and for car washers was 91 EPG (range 4–1,325) (Table 1). None of the participants were coinfected with any of the three soil-transmitted helminths.

**Table 1:** Demographic characteristics of the study participants

Characteristic		Car washers (*N* = 45)	Sand harvesters (*N* = 33)
Age in years, median (range)		27 (18–63)	32 (23–53)
Years worked, median (range)		8 (1–40)	10 (3–30)
Height in centimeters, median (range)		174 (161–187)	174 (157–185)
Weight in kilograms, median (range)		63.6 (46–85.8)	62 (44.4–84.4)
*S. mansoni* infection intensity	Light (1–99 EPG), *n* (%)	33 (73%)	17 (52%)
	Moderate (100–399 EPG), *n* (%)	7 (16%)	11 (33%)
	Heavy (≥400 EPG), *n* (%)	5 (11%)	5 (15%)

### Phenotyping for Treg lymphocyte markers in whole blood by four-color flow cytometry.

**Figure 1.**
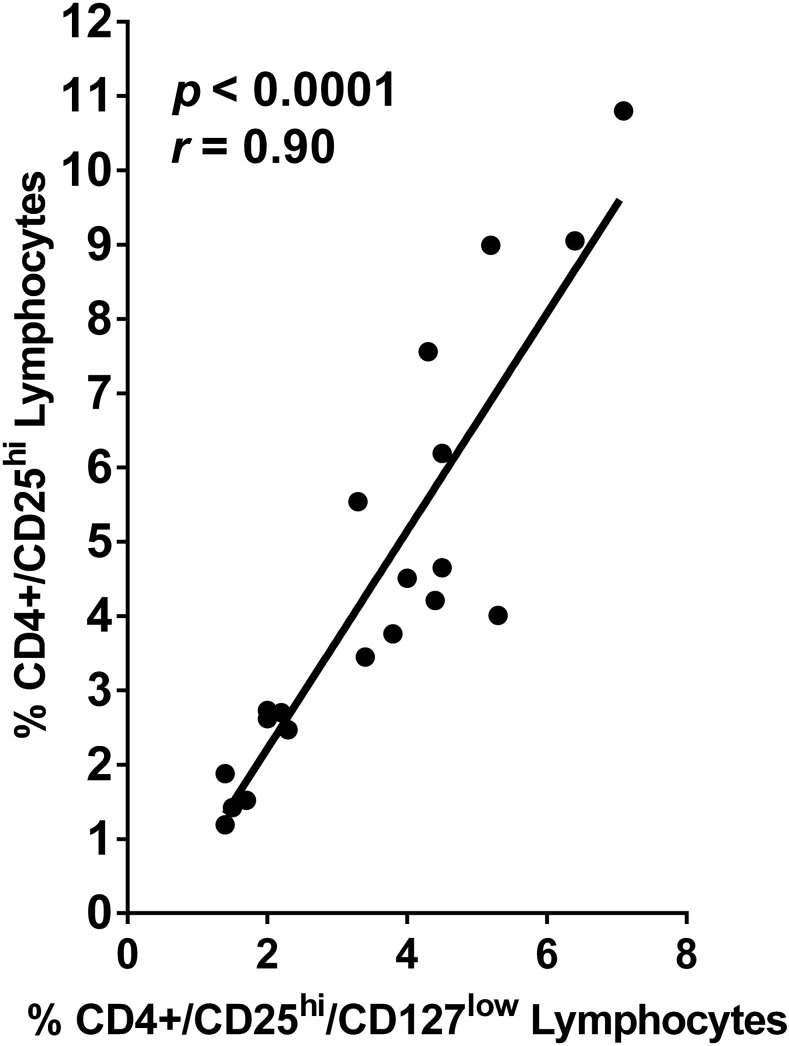
Scatter plot of correlation between the percentages of lymphocytes in peripheral blood mononuclear cells (PBMC) from individuals with schistosomiasis stained for CD4/CD25^hi^ vs. those in the same PBMC population stained with CD4/CD25^hi^/CD127^low^ using Spearman’s correlation test.

Comparing whole blood from subjects with schistosomiasis by direct immunofluorescent staining for expression of CD3, CD4, FoxP3, CD25hi, and CD127, we compared different cell surface marker combinations that have been used to define Treg cells.^9,23,24^ We found that the percentages of CD4+/FoxP3 + lymphocytes did not differ from those of CD4+/CD25^hi^ lymphocytes (*P* = 0.339). Furthermore, linear regression analysis showed that the percentages of CD4+/FoxP3 + and CD4+/CD25^hi^/CD127^low^ lymphocytes in a given person’s peripheral blood correlated (*r* = 0.69; *P* = 0.0007), and the percentages of CD4+/CD25^hi^ cells and CD4+/CD25^hi^/CD127^low^ cells were highly correlated (*r* = 0.90; *P* < 0.0001) (Figure 1). Based on these findings and access to four-color flow cytometry, we have considered that our CD3+/CD4+/CD25^hi^ and CD3+/CD4+/CD25^hi^/CD127^low^ populations represent a reasonable consensus standard set of markers for Treg.^15,24^

### Assessment of the effectiveness of Treg lymphocyte depletion by single-step separation on anti-CD25 magnetic beads.

**Figure 2.**
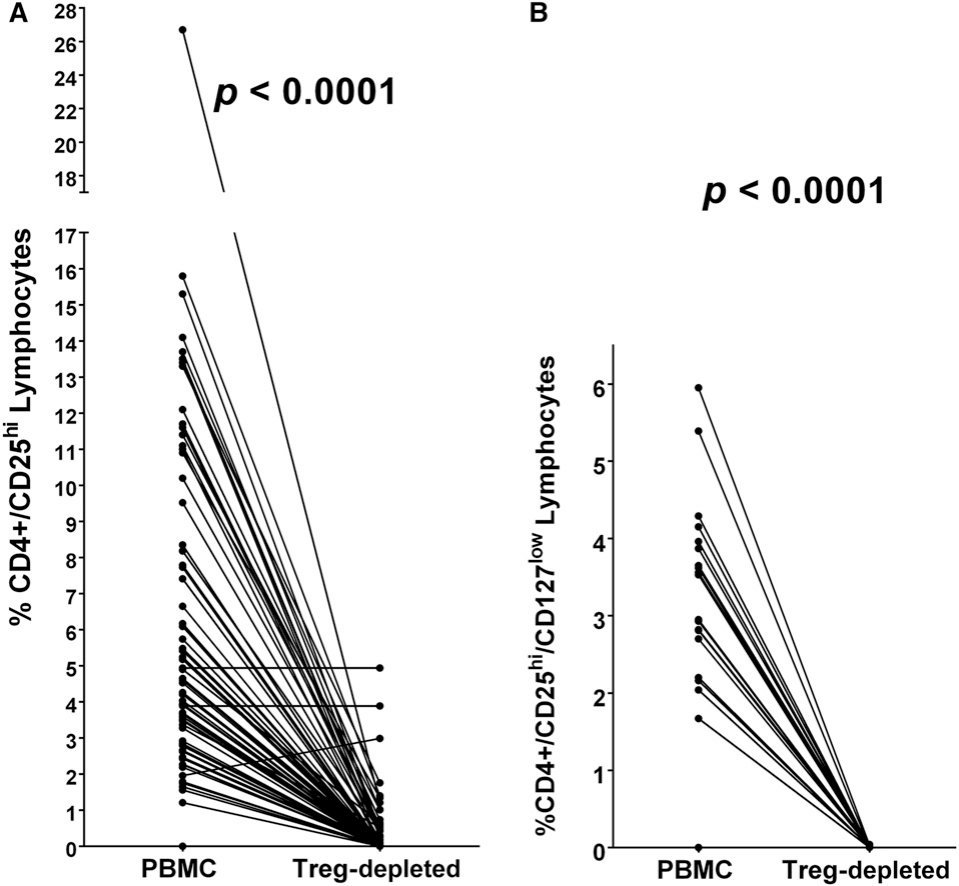
(**A**) Paired analysis of the percentage of lymphocytes in peripheral blood mononuclear cells (PBMC) from individuals with schistosomiasis stained for CD4/CD25hi before and after processing to remove CD25^hi^–positive cells (T regulatory [Treg]-depleted). (**B**) Paired analysis of the percentage of lymphocytes in PBMC from individuals with schistosomiasis stained for CD4/CD25^hi^/CD127^low^ before and after processing to remove CD25hi cells (Treg-depleted). The mean difference between the percentages in the PBMC populations and the Treg-depleted populations were analyzed by Wilcoxon matched-pairs signed rank test and *P* values are indicated above each set of pairs.

We evaluated the ability to selectively deplete Treg cells (CD3+/CD4+/CD25^hi^ cells and CD3+/CD4+/CD25^hi^/CD127^low^ cells) from PBMC preparations with anti-CD25 immunomagnetic bead separation. Flow cytometric analysis of cells before and after separation demonstrated effective removal of CD4+/CD25^hi^ cells from almost all PBMC preparations (Figure 2A) and highly effective removal of CD4+/CD25^hi^/CD127^low^ cells (Figure 2B) from all PBMC preparations.

### Production of IFNγ and IL-10 by PBMCs and following depletion of Treg lymphocytes.

**Figure 3.**
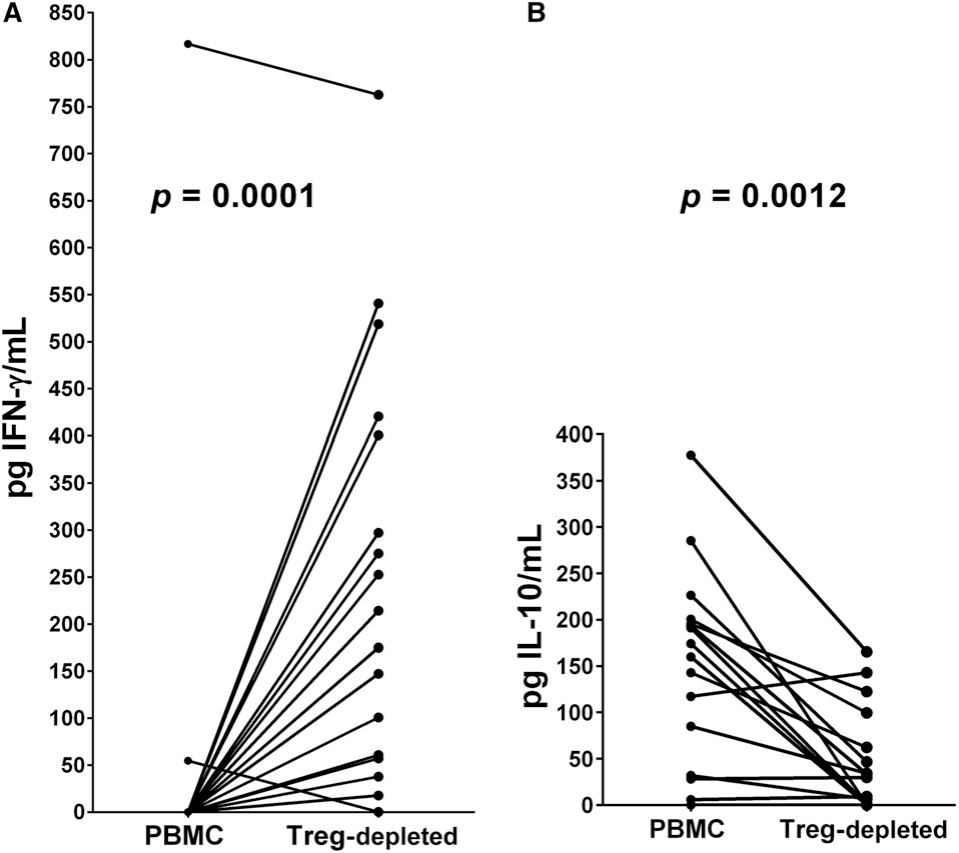
(**A**) Paired analysis of the interferon gamma (IFN-γ) responses of peripheral blood mononuclear cells (PBMC) from individuals with schistosomiasis prior to and after T regulatory (Treg)-depletion in response to phytohemagglutinin (PHA). (**B**) Paired analysis of the interleukin-10 responses of PBMC from individuals with schistosomiasis prior to and after Treg-depletion in response to PHA. Statistical analyses were by the Wilcoxon matched-pairs signed rank test and *P* values are indicated above each set of pairs.

**Figure 4.**
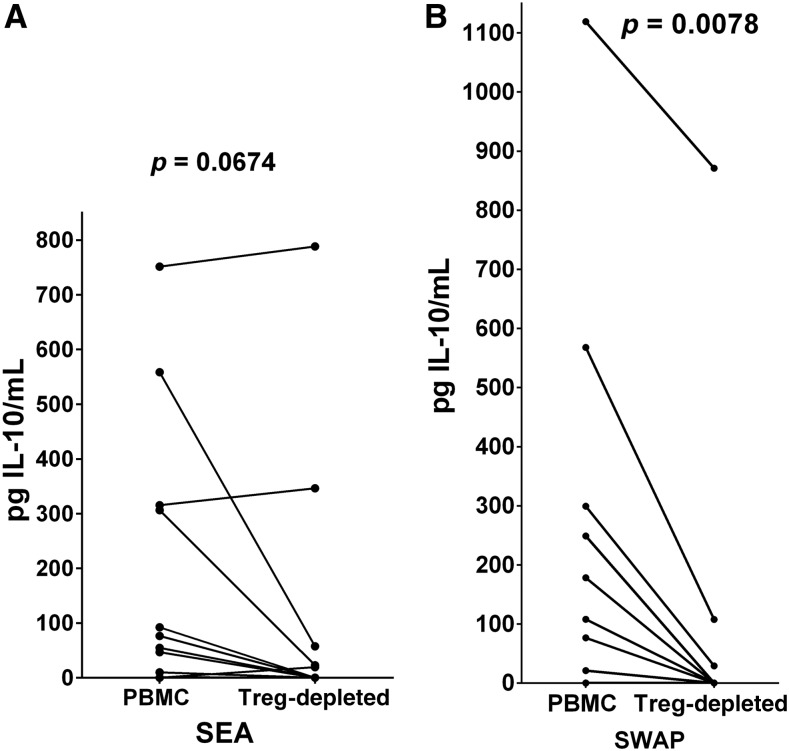
Paired analysis of the interleukin-10 (IL-10) responses of peripheral blood mononuclear cells (PBMC) from individuals with schistosomiasis prior to and after T regulatory (Treg)-depletion in response to (**A**) soluble egg antigens (SEA) and (**B**) soluble worm antigenic preparation (SWAP). Mean SEA-stimulated IL-10 responses were not altered significantly by Treg-depletion (**A**). Soluble worm antigenic preparation-stimulated production of IL-10 (**B**) was significantly decreased by Treg-depletion (*P* = 0.0078). Statistical analyses were by the Wilcoxon matched-pairs signed rank test.

Evaluation of the ability of an individual’s PBMC and Treg-depleted PBMC to produce IFNγ or IL-10 was determined following PHA (3 days) or SEA or SWAP (5 days) stimulation of in vitro cultures. On the appropriate day, culture supernatant fluids were collected, stored at −20°C and later assayed together by cytokine-specific ELISA assays against standard curves. Peripheral blood mononuclear cells from most participants made negligible levels of IFNγ in response to PHA (Figure 3A), but most did produce IL-10 (Figure 3B). However, upon removal of Treg the reverse was true. Treg-depleted PBMC produced significantly more IFNγ (*P* = 0.0001) and significantly less IL-10 (*P* = 0.0012) compared with unseparated cells in response to PHA. The SEA- or SWAP-stimulated IFNγ responses of PBMC were essentially nil and did not change upon removal of Treg (data not shown). Interleukin-10 production in response to SEA was mixed, but responses of most individuals declined following Treg removal (Figure 4A). Soluble worm antigenic preparation stimulation of cultures of Treg-depleted cells demonstrated significantly decreased levels of IL-10 compared with those produced by parallel PBMC cultures (*P* = 0.0078) (Figure 4B), similar to what was seen with PHA stimulation.

### Treg-depletion increases proliferation in response to PHA but not to SEA or SWAP.

**Figure 5.**
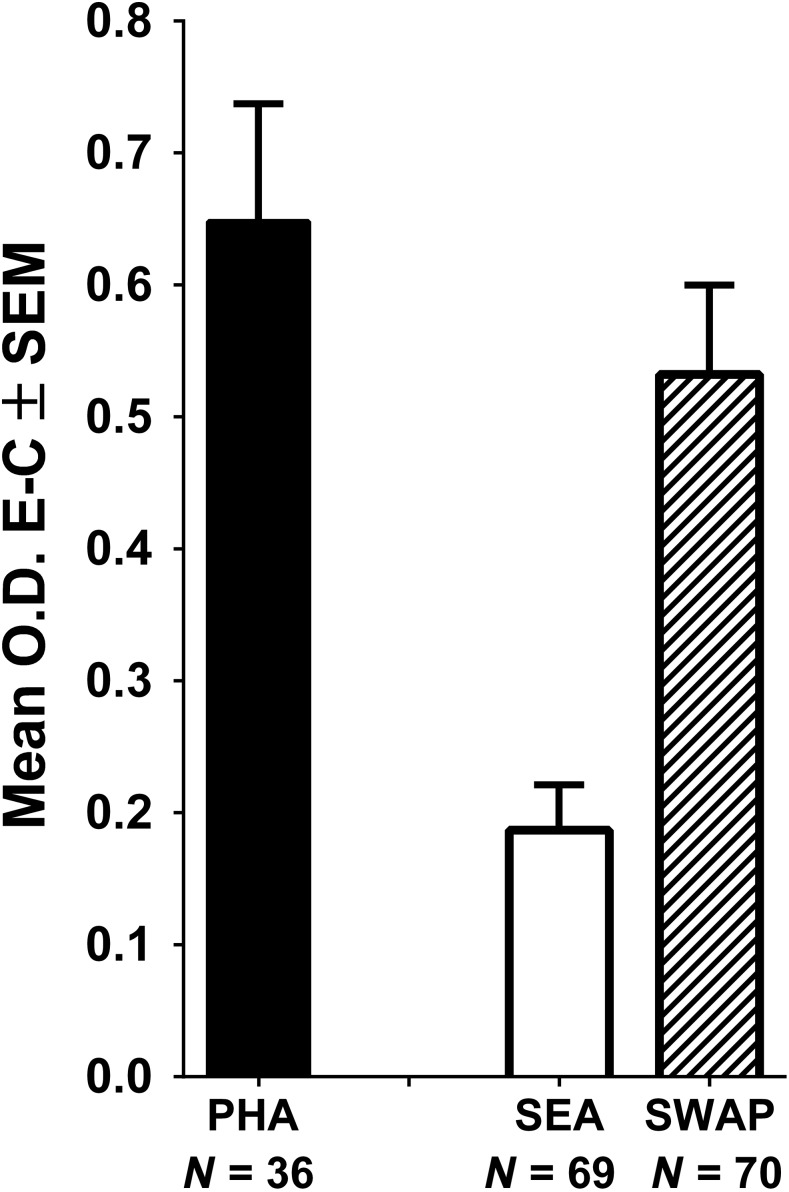
Mean ± SEM proliferative responses of peripheral blood mononuclear cells from individuals with schistosomiasis in response to phytohemagglutinin (PHA) (Day 3 of culture), soluble egg antigen (SEA) or soluble worm antigenic preparation (SWAP) (both Day 5 of culture) as measured by optical density (O.D.) based on BrdU incorporation, minus the incorporation in culture medium alone (PHA, Day 3; SEA and SWAP, Day 5). *N* values indicate the number of individuals contributing to each mean.

**Figure 6.**
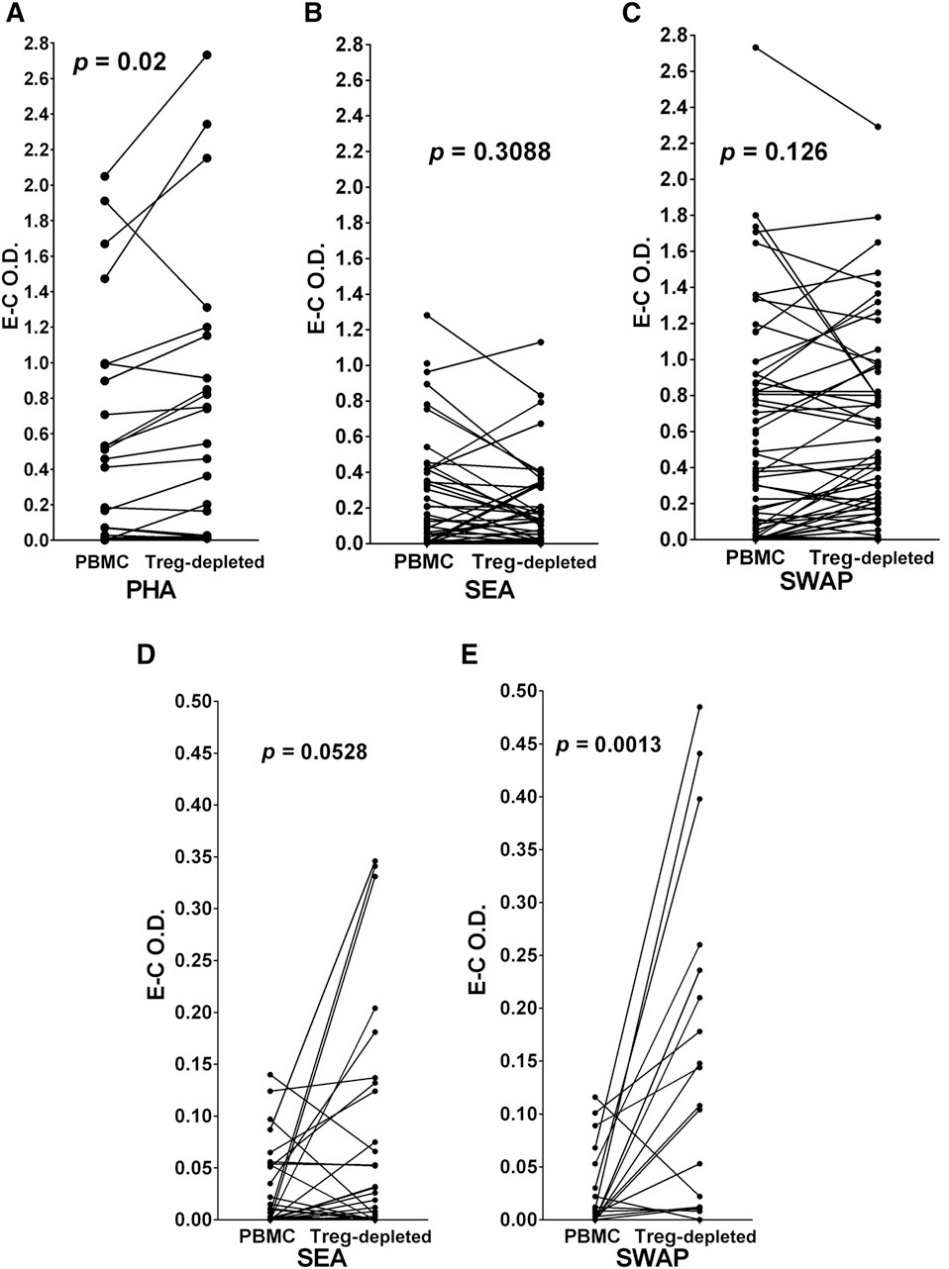
Peripheral blood mononuclear cells (PBMCs) and their parallel T regulatory-depleted populations were cultured in the presence of phytohemagglutinin (PHA), schistosome soluble egg antigen (SEA) or schistosome soluble worm antigenic preparation (SWAP) and their level of proliferation determined by incorporation of BrdU/labeled anti-BrdU as optical density values (O.D.) as Experimental (E; PHA, SEA or SWAP) or Control (C; media alone). Panels are as follows: **A**, PHA; **B**, SEA; **C**, SWAP. Panels **D** and **E** are replotted and reanalyzed from Panels **B** and **C**, respectively, showing the responses of very low (E–C O.D. values < 0.150) responders to SEA or SWAP. E–C O.D. values for each pair are plotted and were analyzed by Wilcoxon matched-pairs signed rank test. *P* values for differences in the mean E–C values are given above each pair in each panel.

Using the BrdU proliferation assay,^25^ we confirmed that optimal responses were obtained at day 3 when PBMC cultures were exposed to the mitogen PHA and by 5 days when cultured with SEA or SWAP. Cultures from those optimum response days are presented in Figure 5. We then compared responsiveness of PBMC and Treg-depleted populations from the same individual to evaluate the effect of Treg removal on PHA-, SEA-, or SWAP-stimulated proliferation. Paired analyses demonstrated that proliferative responses to PHA significantly increased in the Treg-depleted cultures compared with the total PBMC (*P* = 0.02) (Figure 6A). By contrast, Treg depletion did not significantly increase proliferative responses to SEA (*P* = 0.3088) or to SWAP (*P* = 0.126) (Figure 6B and C). When we focused on those participants with very low PBMC responses to antigen (an arbitrary cutoff for very low responses was used; O.D. ≤ 0.150), we observed that Treg removal did result in significantly increased responsiveness to SWAP (*P* = 0.0013, Figure 6D) and marginally increased responses to SEA (*P* = 0.0528, Figure 6E).

### Addition of anti-IL-10 increases PBMC responses, but not Treg-depleted responses to SEA and SWAP.

**Figure 7.**
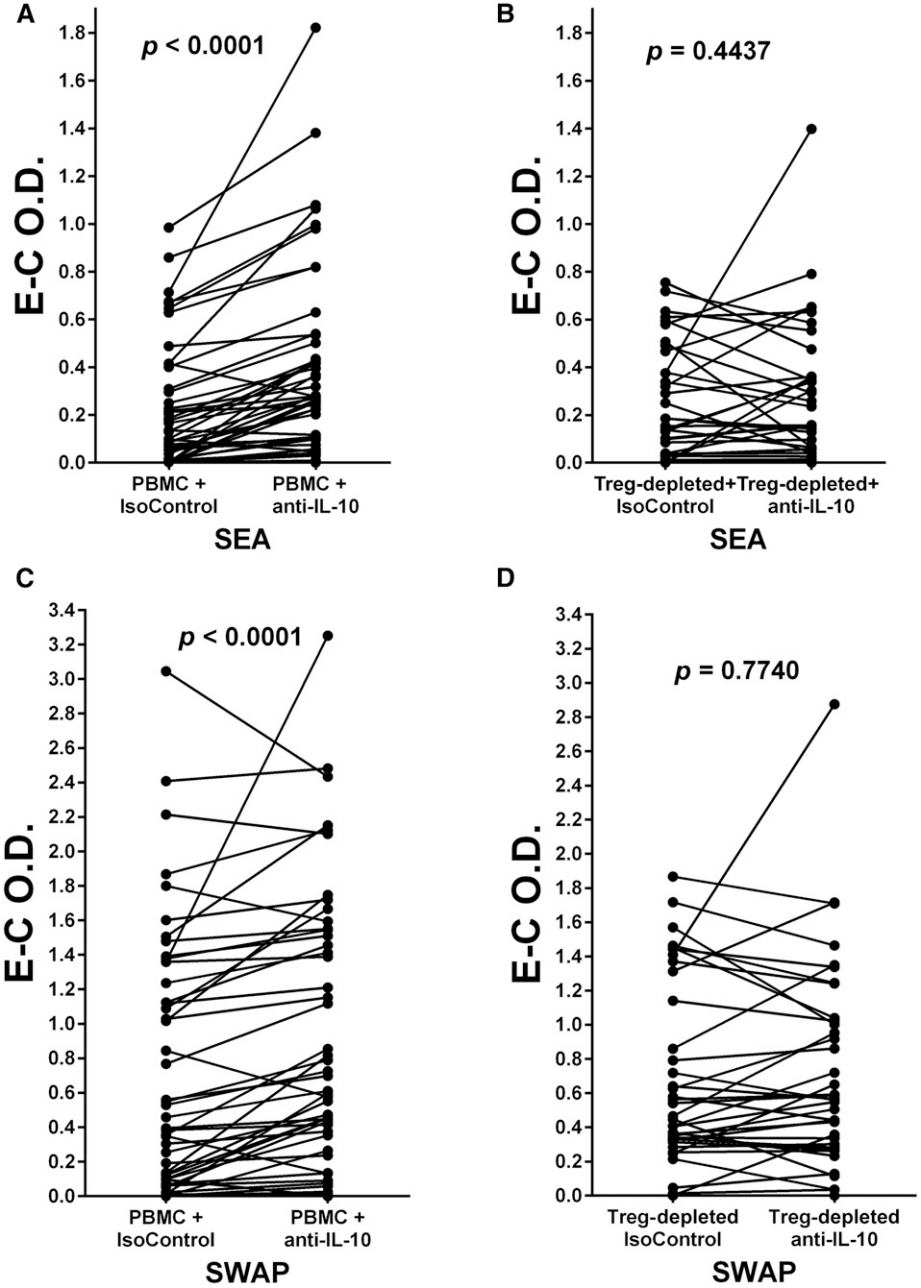
(**A** and **B**) Peripheral blood mononuclear cells and their parallel T regulatory (Treg)-depleted populations were cultured in the presence of schistosome soluble egg antigen (SEA) plus isotype control monoclonal antibody (mAb) (IsoControl) compared with culture in the presence of SEA plus mAb against interleukin-10 (anti-IL-10). (**C** and **D**) Peripheral blood mononuclear cell and their parallel Treg-depleted populations were cultured in the presence of schistosome soluble worm antigenic preparation (SWAP) plus IsoControl compared with culture in the presence of SWAP plus anti-IL-10. The levels of proliferation in the cultures were determined by incorporation of BrdU/labeled anti-BrdU expressed as optical density values (O.D.) as Experimental (E) or Control (C). E–C O.D. values for each pair are plotted and were analyzed by Wilcoxon matched-pairs signed rank test. *P* values for differences in the mean E–C values are given above each pair in each panel.

Addition of monoclonal anti-IL-10, but not a monoclonal isotype control, to parallel cultures of individuals’ PBMC and Treg-depleted cell populations led to increased proliferative responses to both SEA and SWAP by PBMC (*P* < 0.0001 for both), but Treg-depleted populations did not exhibit consistently increased responsiveness (*P* = 0.4437 and *P* = 0.7740, respectively) (Figure 7A–D).

### Addition of anti-TGF-β increases Treg-depleted cell responses to both SEA and SWAP, but only PBMC responses to SEA.

**Figure 8.**
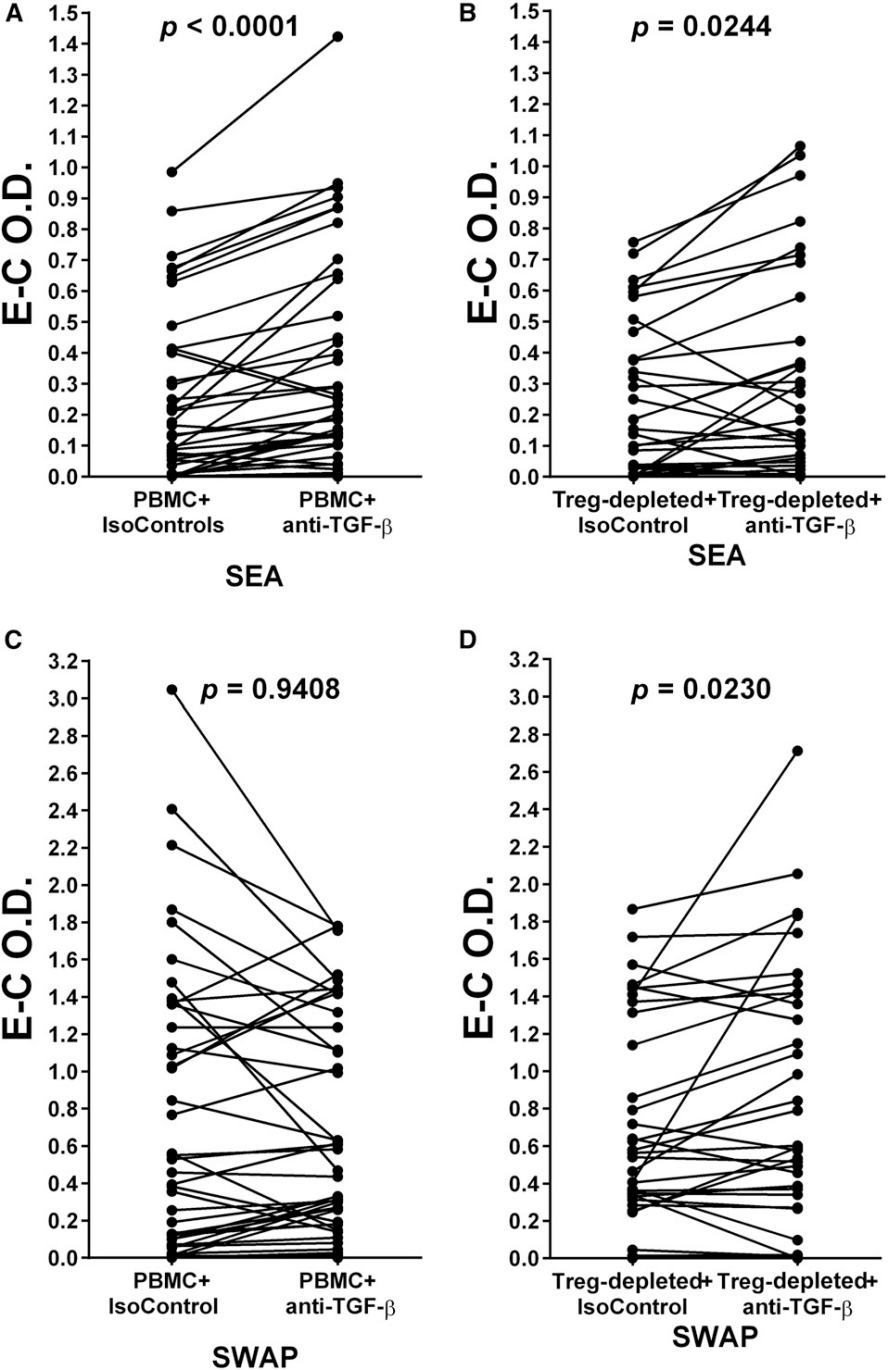
(**A** and **B**) Peripheral blood mononuclear cells and their parallel T regulatory (Treg)-depleted populations were cultured in the presence of schistosome soluble egg antigen (SEA) plus isotype control monoclonal antibody (mAb) (IsoControl) compared with culture in the presence of SEA plus mAb against TGF-β (anti-TGF-β). (**C** and **D**) Peripheral blood mononuclear cell and their parallel Treg-depleted populations were cultured in the presence of schistosome soluble worm antigenic preparation (SWAP) plus IsoControl compared with culture in the presence of SWAP plus anti-TGF-β. The levels of proliferation in the cultures were determined by incorporation of BrdU/labeled anti-BrdU expressed as optical density values (O.D.) as Experimental (E) or Control (C). E–C O.D. values for each pair are plotted and were analyzed by Wilcoxon matched-pairs signed rank test. *P* values for differences in the mean E–C values are given above each pair in each panel.

In contrast to anti-IL-10, addition of monoclonal anti-TGF-β, but not a monoclonal isotype control, to parallel cultures of individuals’ PBMC and Treg-depleted cell populations resulted in increased proliferation to SEA by both a person’s PBMC (*P* < 0.0001) and their parallel cultures of Treg-depleted cells (*P* = 0.0244) (Figure 8A and B). Addition of anti-TGF-β to parallel cultures exposed to SWAP yielded a different pattern of change due to Treg-depletion. Anti-TGF-β failed to consistently alter PBMC responses to SWAP (*P* = 0.9408), but did augment SWAP-stimulated responses by parallel cultures of Treg-depleted cells (*P* = 0.023) (Figure 8C and D).

### Co-addition of anti-IL-10 and anti-TGF-β to cultures of PBMC or Treg-depleted cells yields augmented proliferative responses by both types of cultures to both SEA and SWAP.

**Figure 9.**
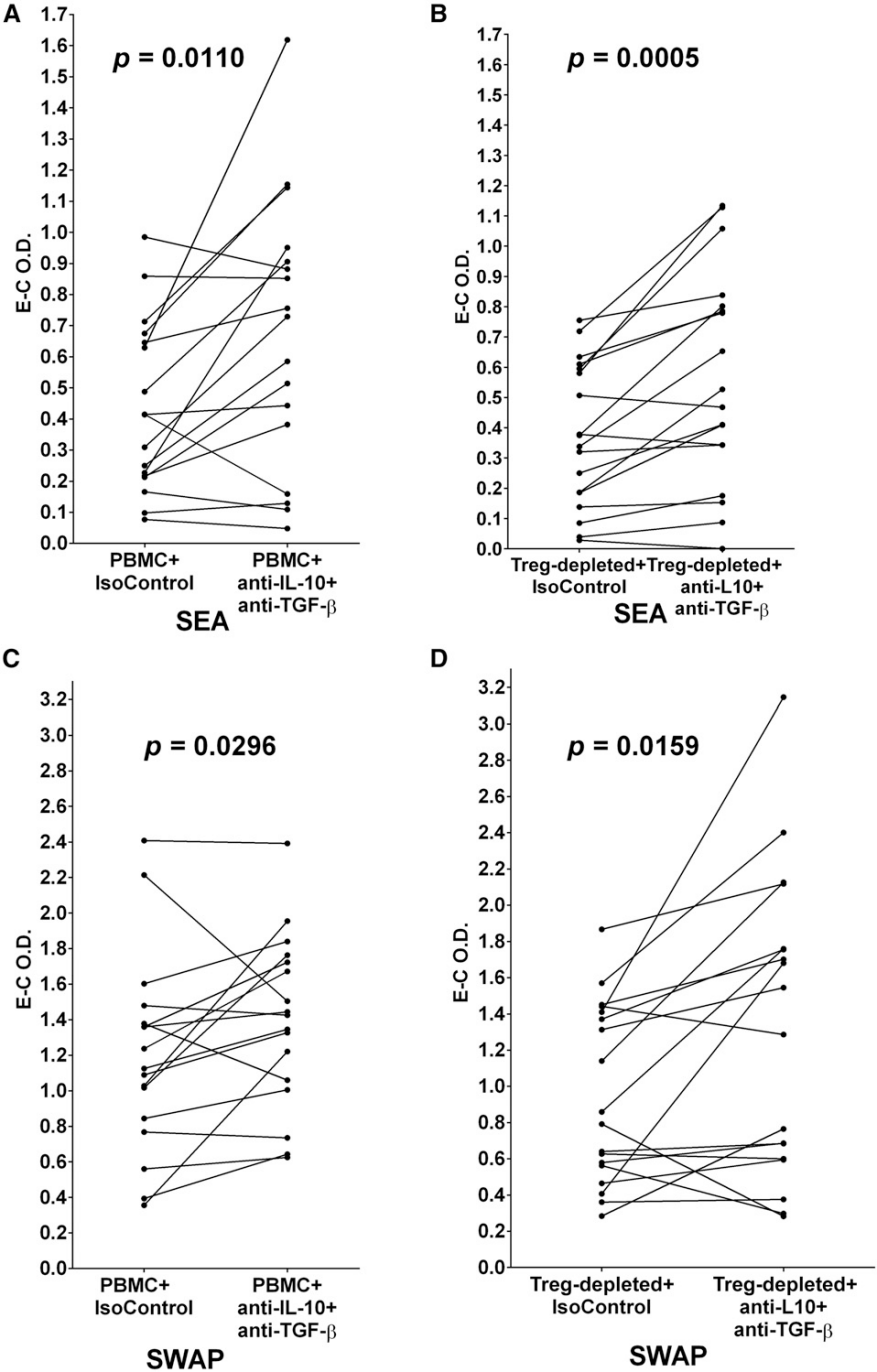
(**A** and **B**) Peripheral blood mononuclear cells and their parallel T regulatory (Treg)-depleted populations were cultured in the presence of schistosome soluble egg antigen (SEA) plus two isotype control monoclonal antibodies (IsoControl) compared with culture in the presence of SEA plus monoclonal antibodies against both interleukin-10 (IL-10) and TGF-β. (**C** and **D**) Peripheral blood mononuclear cell and their parallel Treg-depleted populations were cultured in the presence of schistosome soluble worm antigenic preparation (SWAP) plus two IsoControls compared with culture in the presence of SWAP plus both anti-IL-10 and anti-TGF-β. The levels of proliferation in the cultures were determined by incorporation of BrdU/labeled anti-BrdU expressed as optical density values (O.D.) as Experimental (E) or Control (C). E–C O.D. values for each pair are plotted and were analyzed by Wilcoxon matched-pairs signed rank test. *P* values for differences in the mean E–C values are given above each pair in each panel.

Addition of both anti-IL-10 and anti-TGF-β, but not parallel monoclonal isotype controls, to cultures of individuals’ PBMC and Treg-depleted cells led to increased responses by both types of cultures upon exposure to either SEA or SWAP. This is shown in Figure 9A–D, where PBMC responses to SEA (*P* = 0.0110) and SWAP (*P* = 0.0296) increased as did the proliferative responses of Treg-depleted cultures (*P* = 0.0005 and *P* = 0.0159, respectively).

## Discussion

Schistosomiasis remains a major public health problem in Kenya and many parts of sub-Saharan Africa. National Neglected Tropical Disease programs in most of those countries impacted are making strides to control morbidity.^26–29^ However, reinfection rates after annual mass drug administration (MDA) with PZQ can result in the continued high prevalence of infection. Nevertheless, at least partial resistance to reinfection may occur in some individuals after multiple rounds of treatment and reinfection.^18,30–33^ Understanding human immune responses against schistosomal antigens and their regulation has been the topic of multiple groups and studies,^34^ and elevated levels of Treg has been reported by several studies.^2,4,12,13^ The functional activities of such Treg in human schistosomiasis have been discussed^12^ but remain only partly understood. A continued understanding and characterization of these regulatory cells and their impact on schistosome antigen-specific responses is therefore important for our understanding of this complex infection of millions of people that characteristically presents as a chronic, systemic antigenic exposure.

The current study continues this line of research in regard to Treg-mediated immunoregulation of mitogen-stimulated and schistosomal antigen-specific cytokine production and lymphocyte proliferation in an occupational setting of repeated infections and reinfections. Evidence of possible Treg-mediated Th1 regulation by those harboring schistosomes is seen upon PHA exposure of cultures of PBMC versus Treg-depleted populations, where PBMC from almost all infected individuals fail to produce IFNγ unless Treg are removed (Figure 3A). By contrast, their PBMC responded to PHA by production of IL-10, but this ability was greatly reduced on depletion of Treg, indicating that much of the stimulated IL-10 is likely being produced by Treg (Figure 3B). Neither SEA nor SWAP stimulated IFNγ production by either cell populations, but these antigenic preparations did induce IL-10 production by PBMC cultures, and this ability was decreased in Treg-depleted cultures from most individuals (Figure 4A and B), albeit not significantly in regard to SEA, suggesting that during human schistosomiasis Treg cells are a main source of immunoregulatory IL-10. Interleukin-10 has previously been correlated with control of pathogenesis, reduction of morbidity and prolonged survival in human schistosomiasis,^35^ but has also been linked to a decreased resistance to reinfection.^36^

Peripheral blood mononuclear cell proliferation patterns from these participants in response to PHA, SEA, or SWAP are strikingly similar to those reported previously^21,34,37,38^ where responses to PHA and SWAP are often high, whereas those to SEA are modest or low. Mean PBMC proliferative responses to PHA were augmented by depletion of Treg (Figure 6A), but the overall mean responses to SEA and SWAP were not and expressed considerable heterogeneity (Figure 6B and C). Low responders (selected arbitrarily to have O.D. values ≤ 0.150) to SWAP did increase significantly upon Treg depletion (Figure 6D). However, although most SEA low responders increased with the removal of Treg, the mean of the Treg-depleted responses did not achieve statistical significance (Figure 6E). These results indicate that Treg immunoregulatory activity is more readily apparent in PBMC cultures that respond very poorly to these antigens.

The addition of neutralizing anti-IL-10 to antigen-driven PBMC or whole blood cultures from human schistosome patients has been studied extensively^35,39–43^ and has usually led to increased proliferation or cytokine responses. We also observe that anti-IL-10 mAb is able to alleviate IL-10-mediated immunoregulation of SEA-stimulated proliferation (Figure 7A) and we have extended these observations to show that anti-IL-10 does not change the SEA-stimulated response of Treg-depleted cultures (Figure 7B). The same is true for SWAP-stimulated PBMC or Treg-depleted cultures cocultured with anti-IL-10 (Figure 7C and D). In conjunction with the data in Figure 3B, which showed that depletion of Treg led to a loss of production of IL-10 upon PHA exposure, this finding provides evidence that a major mode of Treg immunoregulation is mediated through the production of IL-10.

The ability of anti-TGF-β mAb to alter the SEA- or SWAP-stimulation of PBMC or Treg-depleted cultures presents a different picture than that with anti-IL-10. Anti-TGF-β again augments the PBMC response to SEA, but also augments the Treg-depleted response to SEA (Figure 8A and B), likely indicating that non-Treg cells are producing much of the immunoregulatory TGF-β. This appears to also be true for SWAP-stimulated Treg cultures (Figure 8D), however the failure of anti-TGF-β to increase SWAP-stimulated proliferation may indicate that the immunoregulatory effect of SWAP-stimulated IL-10 in these cultures is too strong to overcome by blocking with anti-TGF-β. On Treg removal, and with it the source of most of the IL-10 production, anti-TGF-β can effectively block the regulatory TGF-β from non-Treg cells, resulting in responsiveness.

When anti-IL-10 mAb and anti-TGF-β mAb are both added to SEA-stimulated PBMC or Treg-depleted cultures the mean proliferative responses of both types of cultures are significantly increased (Figure 9A and B). The same is true when this combinatorial blockade is included in SWAP-stimulated PBMC or Treg-depleted cultures (Figure 9C and D). It is apparent that blockade of both immunoregulatory cytokines, IL-10 and TGF-β, is sufficient to allow augmented antigen-specific responses to both SEA and SWAP by cells from most infected individuals, and in some cases the increases are quite substantial. Other studies have also reported that Treg function through the production of anti-inflammatory cytokines, for example, IL-10 and/or TGF-β in a variety of immune-mediated conditions.^44–47^

Immunoregulation is an integral part of chronic human schistosomiasis.^34^ Elevated Treg and the involvement of IL-10^4,12^ and even TGF-β^48–50^ have been reported in studies of people with schistosomiasis or other chronic helminthic infections. However, the antigen-specific functional abilities of the elevated Treg populations in persons with schistosomiasis have not been generally reported. Here we provide evidence that Treg, IL-10, and TGF-β are involved in the regulation of SEA and SWAP responses. It appears that Treg produce much of the IL-10 stimulated by either of the antigens, although the source of at least some of the TGF-β remains unclear. Other possible sources of antigen-stimulated TGF-β have been reported and include epithelial cells, fibroblasts, and immune-associated cells such as macrophages and eosinophils.^51^ Regulatory B cells from schistosome patients also produce IL-10^52^ as can CD8 + cells IL-10^53^ and should perhaps be further investigated in schistosomiasis. Regulation of responses to SEA may be responsible for the control of morbidity caused by granulomatous reactions to those schistosome eggs that fail to be excreted and become lodged in the tissues.^34^ The chronic infection induced–immunoregulatory mechanisms demonstrated here, Treg, IL-10 and TGF-β may, therefore, curtail severe disease in the majority of individuals infected with *S. mansoni* and other helminthic infections.^6,34,54^ It is also possible that these same immunoregulatory mechanisms, when generated in response to SWAP may participate in regulation of otherwise protective immune responses against multiple reinfections.^36,55^

## References

[1] Colley DG Bustinduy AL Secor WE King CH, 2014 Human schistosomiasis. Lancet 383: 2253–2264. 24698483 10.1016/S0140-6736(13)61949-2 PMC4672382

[2] Romano A 2016 FOXP3+ regulatory T cells in hepatic fibrosis and splenomegaly caused by *Schistosoma japonicum*: the spleen may be a major source of Tregs in subjects with splenomegaly. PLoS Negl Trop Dis 10: e0004306. 26731721 10.1371/journal.pntd.0004306 PMC4701139

[3] Wilson S Jones FM Kenty LC Mwatha JK Kimani G Kariuki HC Dunne DW, 2014 Posttreatment changes in cytokines induced by *Schistosoma mansoni* egg and worm antigens: dissociation of immunity- and morbidity-associated type 2 responses. J Infect Dis 209: 1792–1800. 24357629 10.1093/infdis/jit826 PMC4017363

[4] Nausch N Midzi N Mduluza T Maizels RM Mutapi F, 2011 Regulatory and activated T cells in human *Schistosoma haematobium* infections. PLoS One 6: e16860. 21347311 10.1371/journal.pone.0016860 PMC3037381

[5] Joseph S 2004 Increases in human T helper 2 cytokine responses to *Schistosoma mansoni* worm and worm‐tegument antigens are induced by treatment with praziquantel. J Infect Dis 190: 835–842. 15272413 10.1086/422604

[6] Silveira AM 2004 Human schistosomiasis mansoni: intensity of infection differentially affects the production of interleukin-10, interferon-gamma and interleukin-13 by soluble egg antigen or adult worm antigen stimulated cultures. Trans R Soc Trop Med Hyg 98: 514–519. 15251399 10.1016/j.trstmh.2003.11.009

[7] Roberts M Butterworth AE Kimani G Kamau T Fulford AJ Dunne DW Ouma JH Sturrock RF, 1993 Immunity after treatment of human schistosomiasis: association between cellular responses and resistance to reinfection. Infect Immun 61: 4984–4993. 8225573 10.1128/iai.61.12.4984-4993.1993 PMC281273

[8] Rodríguez-Perea AL Arcia ED Rueda CM Velilla PA, 2016 Phenotypical characterization of regulatory T cells in humans and rodents. Clin Exp Immunol 185: 281–291. 27124481 10.1111/cei.12804 PMC4991523

[9] Miyara M Sakaguchi S, 2011 Human FoxP3(+) CD4(+) regulatory T cells: their knowns and unknowns. Immunol Cell Biol 89: 346–351. 21301480 10.1038/icb.2010.137

[10] Roncador G 2005 Analysis of FOXP3 protein expression in human CD4+CD25+ regulatory T cells at the single-cell level. Eur J Immunol 35: 1681–1691. 15902688 10.1002/eji.200526189

[11] Sakaguchi S, 2000 Regulatory T cells: key controllers of immunologic self-tolerance. Cell 101: 455–458. 10850488 10.1016/s0092-8674(00)80856-9

[12] Schmiedel Y Mombo-Ngoma G Labuda LA Janse JJ de Gier B Adegnika AA Issifou S Kremsner PG Smits HH Yazdanbakhsh M, 2015 CD4+CD25hiFOXp3+ regulatory T cells and cytokine responses in human schistosomiasis before and after treatment with praziquantel. PLoS Negl Trop Dis 9: e0003995. 26291831 10.1371/journal.pntd.0003995 PMC4546370

[13] Watanabe K Mwinzi PNM Black CL Muok EMO Karanja DMS Secor WE Colley DG, 2007 T regulatory cell levels decrease in people infected with *Schistosoma mansoni* on effective treatment. Am J Trop Med Hyg 77: 676–682. 17978070 PMC2602861

[14] Roncarolo MG Gregori S, 2008 Is FOXP3 a bona fide marker for human regulatory T cells? Eur J Immunol 38: 925–927. 18395862 10.1002/eji.200838168

[15] Hartigan-O’Connor DJ Poon C Sinclair E McCune JM, 2007 Human CD4+ regulatory T cells express lower levels of the IL-7 receptor alpha chain (CD127), allowing consistent identification and sorting of live cells. J Immunol Methods 319: 41–52. 17173927 10.1016/j.jim.2006.10.008

[16] Black CL Mwinzi PNM Muok EMO Abudho B Fitzsimmons CM Dunne DW Karanja DMS Secor WE Colley DG, 2010 Influence of exposure history on the immunology and development of resistance to human Schistosomiasis mansoni. PLoS Negl Trop Dis 4: e637. 20351784 10.1371/journal.pntd.0000637 PMC2843635

[17] Mwinzi PNM Ganley-Leal L Black CL Secor WE Karanja DMS Colley DG, 2009 Circulating CD23+ B cell subset correlates with the development of resistance to *Schistosoma mansoni* reinfection in occupationally exposed adults who have undergone multiple treatments. J Infect Dis 199: 272–279. 19072134 10.1086/595792 PMC2636678

[18] Karanja DMS Hightower AW Colley DG Mwinzi PNM Galil K Andove J Secor WE, 2002 Resistance to reinfection with *Schistosoma mansoni* in occupationally exposed adults and effect of HIV-1 co-infection on susceptibility to schistosomiasis: a longitudinal study. Lancet 360: 592–596. 12241930 10.1016/S0140-6736(02)09781-7

[19] Katz N Chaves A Pellegrino J, 1972 A simple device for quantitative stool thick-smear technique in Schistosomiasis mansoni. Rev Inst Med Trop São Paulo 14: 397–400. 4675644

[20] WHO, 2013 *Schistosomiasis: Progress Report 2001–2011, Strategic Plan 2012–2020*. Geneva, Switzerland: World Health Organization.

[21] Colley DG Cook JA Freeman GL Bartholomew RK Jordan P, 1977 Immune responses during human schistosomiasis mansoni: I. In vitro lymphocyte blastogenic responses to heterogeneous antigenic preparations from schistosome kggs, worms and cercariae. Int Arch Allergy Immunol 53: 420–433. 300716

[22] Boros DL Warren KS, 1970 Delayed hypersensitivity-type granuloma formation and dermal reaction induced and elicited by a soluble factor isolated from *Schistosoma mansoni* eggs. J Exp Med 132: 488–507. 5535626 10.1084/jem.132.3.488 PMC2138804

[23] Sakaguchi S Wing K Miyara M, 2007 Regulatory T cells—a brief history and perspective. Eur J Immunol 37 (Suppl 1): S116–S123. 17972355 10.1002/eji.200737593

[24] Schmetterer KG Neunkirchner A Pickl WF, 2012 Naturally occurring regulatory T cells: markers, mechanisms, and manipulation. FASEB J 26: 2253–2276. 22362896 10.1096/fj.11-193672

[25] Dock J Hultin L Hultin P Elliot J Yang OO Anton PA Jamieson BD Effros RB, 2017 Human immune compartment comparisons: optimization of proliferative assays for blood and gut T lymphocytes. J Immunol Methods 445: 77–87. 28336395 10.1016/j.jim.2017.03.014 PMC5505254

[26] Ndayishimiye O Ortu G Soares Magalhaes RJ Clements A Willems J Whitton J Lancaster W Hopkins A Fenwick A, 2014 Control of neglected tropical diseases in Burundi: partnerships, achievements, challenges, and lessons learned after four years of programme implementation. PLoS Negl Trop Dis 8: e2684. 10.1371/journal.pntd.0002684 PMC4006741 24785993

[27] Hanson C Weaver A Zoerhoff KL Kabore A Linehan M Doherty A Engels D Savioli L Ottesen EA, 2012 Integrated implementation of programs targeting neglected tropical diseases through preventive chemotherapy: identifying best practices to roll out programs at national scale. Am J Trop Med Hyg 86: 508–513. 22403327 10.4269/ajtmh.2012.11-0589 PMC3284372

[28] Zhang Y MacArthur C Mubila L Baker S, 2010 Control of neglected tropical diseases needs a long-term commitment. BMC Med 8: 67. 21034473 10.1186/1741-7015-8-67 PMC2987894

[29] Fenwick A 2009 The schistosomiasis control initiative (SCI): rationale, development and implementation from 2002–2008. Parasitology 136: 1719–1730. 19631008 10.1017/S0031182009990400

[30] De Moira AP Jones FM Wilson S Tukahebwa E Fitzsimmons CM Mwatha JK Bethony JM Kabatereine NB Dunnea DW, 2013 Effects of treatment on IgE responses against parasite allergen-like proteins and immunit to reinfection in childhood schistosome and hookworm coinfections. Infect Immun 81: 23–32. 23071136 10.1128/IAI.00748-12 PMC3536155

[31] Bourke CD Nausch N Rujeni N Appleby LJ Mitchell KM Midzi N Mduluza T Mutapi F, 2013 Integrated analysis of innate, Th1, Th2, Th17, and regulatory cytokines identifies changes in immune polarisation following treatment of human schistosomiasis. J Infect Dis 208: 159–169. 23045617 10.1093/infdis/jis524 PMC3666130

[32] Wilson MS Cheever AW White SD Thompson RW Wynn TA, 2011 IL-10 blocks the development of resistance to re-infection with *Schistosoma mansoni*. PLoS Pathog 7: e1002171. 21829367 10.1371/journal.ppat.1002171 PMC3150278

[33] Vereecken K Naus CWA Polman K Scott JT Diop M Gryseels B Kestens L, 2007 Associations between specific antibody responses and resistance to reinfection in a Senegalese population recently exposed to *Schistosoma mansoni*. Trop Med Int Health 12: 431–444. 17313515 10.1111/j.1365-3156.2006.01805.x

[34] Colley DG Secor WE, 2014 Immunology of human schistosomiasis. Parasite Immunol 36: 347–357. 25142505 10.1111/pim.12087 PMC4278558

[35] Corrêa-Oliveira R 1998 Cytokines as determinants of resistance and pathology in human *Schistosoma mansoni* infection. Braz J Med Biol Res 31: 171–177. 9686196 10.1590/s0100-879x1998000100024

[36] van den Biggelaar AHJ Borrmann S Kremsner P Yazdanbakhsh M, 2002 Immune responses induced by repeated treatment do not result in protective immunity to *Schistosoma haematobium*: interleukin (IL)-5 and IL-10 responses. J Infect Dis 186: 1474–1482. 12404164 10.1086/344352

[37] Gazzinelli G Montesano M, 1987 Immune response in different clinical groups of schistosomiasis patients. Memórias do Inst 82 (Suppl 4): 95–100. 10.1590/s0074-02761987000800015 3151120

[38] Colley DG Garcia AA Lambertucci JR Parra JC Katz N Rocha RS Gazzinelli G, 1986 Immune responses during human schistosomiasis. XII. Differential responsiveness in patients with hepatosplenic disease. Am J Trop Med Hyg 35: 793–802. 3089040

[39] Montenegro SM 1999 Cytokine production in acute versus chronic human Schistosomiasis mansoni: the cross-regulatory role of interferon-gamma and interleukin-10 in the responses of peripheral blood mononuclear cells and splenocytes to parasite antigens. J Infect Dis 179: 1502–1514. 10228073 10.1086/314748

[40] Grogan JL Kremsner PG Deelder AM Yazdanbakhsh M, 1998 The effect of anti-IL-10 on proliferation and cytokine production in human schistosomiasis: fresh versus cryopreserved cells. Parasite Immunol 20: 345–349. 9717196 10.1046/j.1365-3024.1998.00157.x

[41] Malaquias LC Falcão PL Silveira AM Gazzinelli G Prata A Coffman RL Pizziolo V Souza CP Colley DG Correa-Oliveira R, 1997 Cytokine regulation of human immune response to *Schistosoma mansoni*: analysis of the role of IL-4, IL-5 and IL-10 on peripheral blood mononuclear cell responses. Scand J Immunol 46: 393–398. 9350291 10.1046/j.1365-3083.1997.d01-136.x

[42] Araújo M 1996 Evidence of a T helper type 2 activation in human schistosomiasis. Eur J Immunol 26: 1399–1403. 8647223 10.1002/eji.1830260633

[43] King CL 1996 Cytokine control of parasite-specific anergy in human urinary schistosomiasis. IL-10 modulates lymphocyte reactivity. J Immunol 156: 4715–4721. 8648117

[44] Tran DQ, 2012 TGF-β: the sword, the wand, and the shield of FOXP3(+) regulatory T cells. J Mol Cell Biol 4: 29–37. 22158907 10.1093/jmcb/mjr033

[45] Kochetkova I Thornburg T Callis G Pascual DW, 2011 Segregated regulatory CD39+CD4+ T cell function: TGF-β-producing Foxp3- and IL-10-producing Foxp3+ cells are interdependent for protection against collagen-induced arthritis. J Immunol 187: 4654–4666. 21967895 10.4049/jimmunol.1100530 PMC3237119

[46] Hussain S Paterson Y, 2004 CD4+CD25+ regulatory T cells that secrete TGFβ and IL-10 are preferentially induced by a vaccine vector. J Immunother 27: 339–346. 15314542 10.1097/00002371-200409000-00002

[47] Zhang X Koldzic DN Izikson L Reddy J Nazareno RF Sakaguchi S Kuchroo VK Weiner HL, 2004 IL-10 is involved in the suppression of experimental autoimmune encephalomyelitis by CD25+CD4+ regulatory T cells. Int Immunol 16: 249–256. 14734610 10.1093/intimm/dxh029

[48] Li MO Wan YY Sanjabi S Robertson AK Flavell RA, 2006 Transforming growth factor-beta regulation of immune responses. Annu Rev Immunol 24: 99–146. 16551245 10.1146/annurev.immunol.24.021605.090737

[49] Omer FM Kurtzhals JAL Riley EM, 2000 Maintaining the immunological balance in parasitic infections: a role for TGF-β? Parasitol Today 16: 18–23. 10637583 10.1016/s0169-4758(99)01562-8

[50] Reed SG, 1999 TGF-beta in infections and infectious diseases. Microbes Infect 1: 1313–1325. 10611760 10.1016/s1286-4579(99)00252-x

[51] Akdis M 2016 Interleukins (from IL-1 to IL-38), interferons, transforming growth factor β, and TNF-α: receptors, functions, and roles in diseases. J Allergy Clin Immunol 138: 984–1010. 27577879 10.1016/j.jaci.2016.06.033

[52] van der Vlugt LE Zinsou JF Ozir-Fazalalikhan A Kremsner PG Yazdanbakhsh M Adegnika AA Smits HH, 2014 Interleukin 10 (IL-10)–producing CD1dhi regulatory B cells from *Schistosoma haematobium*–infected individuals induce IL-10–positive T cells and suppress effector T-cell cytokines. J Infect Dis 210: 1207–1216. 24795476 10.1093/infdis/jiu257

[53] Leavy O, 2010 Regulatory T cells: CD8+ TReg cells join the fold. Nat Rev Immunol 10: 680–681. 10.1038/nri2862 20879168

[54] Colley DG Barsoum IS Dahawi HSS Gamil F Habib M El Alamy MA, 1986 Immune responses and immunoregulation in relation to human schistosomiasis in Egypt. III. Immunity and longitudinal studies of in vitro responsiveness after treatment. Trans R Soc Trop Med Hyg 80: 952–957. 3111030 10.1016/0035-9203(86)90268-3

[55] Oliveira LFA Moreno EC Gazzinelli G Silveira AMS Gazzinelli A Loverde P Leite PM, 2006 Cytokine production associated with periportal fibrosis during chronic schistosomiasis mansoni in humans. Infect Immun 74: 1215–1221. 16428771 10.1128/IAI.74.2.1215-1221.2006 PMC1360316

